# LONELINESS AND HEALTH AMONG OLDER ADULTS: RESULTS FROM THE UNIVERSITY OF MICHIGAN NATIONAL POLL ON HEALTHY AGING

**DOI:** 10.1093/geroni/igz038.2233

**Published:** 2019-11-08

**Authors:** Erica Solway, John Piette, Matthias Kirch, Dianne Singer, Jeffrey Kullgren, Preeti Malani

**Affiliations:** 1 Institute for Healthcare Policy and Innovation, University of Michigan, Ann Arbor, Michigan, United States; 2 University of Michigan, Ann Arbor, Michigan, United States

## Abstract

In October 2018, the University of Michigan National Poll on Healthy Aging conducted an online survey using a nationally representative household sample of adults age 50 to 80. One in three respondents (34%) reported feeling a lack of companionship and 27% reported feeling isolated from others some of the time or often during the past year. Those with fair or poor self-reported physical health, mental health, or hearing were more likely to report feeling a lack of companionship or feel isolated as were those who reported less frequently engaging in healthy behaviors. More than one in four (28%) reported social contact with people outside of their household once a week or less. Given the high prevalence of loneliness and its connection to poor health and health behaviors, research on this important issue and efforts to increase social engagement among older adults deserve increased attention.

